# Effect of Phone-Based Enhanced Adherence Counseling (EAC) Among Virally Unsuppressed Key Population (KP)

**DOI:** 10.7759/cureus.38005

**Published:** 2023-04-23

**Authors:** Courage Ekejiuba, Terfa Timbri, Amara Frances Chizoba, Ololade Dalley, Utsav Gurjar, Gloria T Ekejiuba, Victor Enejoh, Olanrewaju Olayiwola, John Okpanachi Oko, Amana Effiong, Ugochinyere Ikechukwu, Chikaodili Udegbunam, Lovette Oji, Okelue E Okobi

**Affiliations:** 1 Preventive Medicine, Excellence Community Education Welfare Scheme, Abuja, NGA; 2 Monitoring and Evaluation, Caritas Nigeria, Abia, NGA; 3 Research, Renewal Research Institute, Houston, USA; 4 Geriatrics, Mission to Elderlies Foundation, Awka, NGA; 5 Dermatology, American University of St Vincent, Kingstown, VCT; 6 Internal Medicine, Caribbean Medical University-School of Medcine, Wilemstied, CUW; 7 Dentistry, National Hospital, Abuja, NGA; 8 Preventive Medicine, Caritas Nigeria, Abuja, NGA; 9 Family Medicine, Windsor University School of Medicine, St. Kitts, KNA; 10 Radiodiagnosis, Alex Ekwueme Federal University Teaching Hospital, Abakaliki, NGA; 11 Internal Medicine, Abia State University, Uturu, New Jersey, USA; 12 Family Medicine, Medficient Health Systems, Laurel, USA; 13 Family Medicine, Lakeside Medical Center, Belle Glade, USA

**Keywords:** phone based counseling, hiv counseling, viral suppression, enhanced adherence counselling (eac), people living with hiv/aids, hiv viral load, " "men who have sex with other men" (msm), hiv aids

## Abstract

Background: Despite the reduced human immunodeficiency virus (HIV) disease burden in Nigeria and globally, the key populations (KPs) can be disproportionately burdened with HIV infection and lower treatment coverage and outcome. A viral load (VL) test is needed to monitor the treatment outcome of KP with VL suppression of < 1000 copies/mL, demonstrating a positive treatment outcome. For unsuppressed VL, enhanced adherence counseling (EAC) may improve viral suppression in people living with HIV/KPs living with HIV (PLHIV/KPLHIV). Conventionally, EAC sessions are done for 3 months through physical visits. Due to the challenges of monthly visits (including transportation, socioeconomic status, and high mobility among KPs), other EAC delivery models need to be explored. We aimed to assess the effect of phone EAC sessions among virally unsuppressed KPs compared to physical EAC.

Method: Using a prospective intervention study design with a sample size of 484, unsuppressed KPLHIV in Delta State Nigeria were selectively stratified (non-randomized) using a simple stratification (ability vs. inability to physically attend EAC sessions in-person) into an intervention group and a control group, receiving phone-based EAC sessions and physical EAC sessions respectively. Repeated VL tests were done 3 months after the intervention, and viral suppression was pegged at the WHO recommendation of <1000 copies/mL. The SPSS version 24.0 (SPSS Inc., Chicago, USA) was used for data analysis of variables within and between study groups. Significance was interpreted at p < 0.05.

Result: Participants were 87.4% males {out of which 75.0% (363/484) identified as men who have sex with men (MSM)} with a mean age of 26 ± 2 years. The intervention group had a slightly higher EAC completion rate at 99.6% than the control group (97.9%). Both groups showed significant differences in viral suppression from 0% to a mean suppression of 88.7% with p < 0.01. The intervention group achieved better suppression (90.5%) than the control group (86.7%).

Conclusion: EAC effectively achieves viral suppression by up to 90% among KPLHIV. Phone-based EAC has also proven effective and, in our findings, slightly more effective than the conventional physical EAC and is recommended among KPLHIV with the known challenge of transportation or poor mobility.

## Introduction

Nigeria has the fourth largest human immunodeficiency virus (HIV) epidemic in the world, with approximately 1.8 million people living with HIV and national viral suppression at 72% in 2020 [1-3]. In the past decades, there has been improvement in HIV epidemic score cards in Nigeria but despite the reduction in HIV disease burden, decline in HIV incidence and expanded coverage of antiretroviral therapy (ART) in Nigeria and on global scale, HIV is not equally distributed across populations [4-6]. Given the biology of HIV and individual, network-level and structural risk determinants for infection, key populations (KPs) have been found to be disproportionately at risk for HIV infection [7]. KPs who are disproportionately at risk for HIV infection include men who have sex with men (MSM), transgender women, sex workers especially female sex workers (FSW), people who inject drugs (PWID), and incarcerated populations [8]. Despite control measures being put in place for the control of the virus in the country, a huge gap still exists in the national response to KPs [6, 9-11], thus the gains in HIV epidemic control have not been uniform. For effective and efficient control, therefore, the KP gaps must be bridged. Lack of proper planning with innovative approaches to reach and cater for KP can, therefore, make achieving HIV epidemic control an uphill task as they will continue to be a reservoir of new HIV infections [6].

Viral load (VL) testing has been established and empirically supported as a key strategy for the effective management of HIV across various climes. It is the single most important laboratory monitoring tool for effective long-term treatment strategies in low-income settings and, after initial screening (where resources are limited), it is considered a priority over CD4 determination. In the KP program the VL test is needed to monitor treatment outcomes in HIV program. Specifically, routine VL monitoring can serve as a metric of optimal adherence to ART and an indicator of potential ART resistance, thereby serving as a means of gauging treatment response. Apart from individual-level benefits, VL monitoring can also be a population-level HIV surveillance tool and a measure of programmatic success even among KP [4]. VL suppression of less than 1000 copies/mL demonstrates a positive treatment outcome among PLHIV and in advent of viral un-suppression, enhanced adherence counseling (EAC) is provided and reasons for viral un-suppression are identified and addressed [11-12].

The WHO, therefore, recommends EAC to improve treatment outcomes in PLHIV. EAC is an interventional program that provides targeted adherence counseling for unsuppressed VL among people living with HIV who are receiving ART before diagnosing treatment failure [12]. Some of the components of this counseling are to assess the requirements, provide adherence counseling, educational sessions, and follow-up sessions [13-14]. Studies have shown that some of the common factors associated with low viral suppression were knowledge and perceptions about anti-retroviral therapy (ART) especially negative perceptions and poor knowledge about ART are important intrapersonal barriers for adherence [15-16]. In addition, due to poor socioeconomic status, some KP clients are known for high mobility and thus may be unsettled to have time to receive education on ART for improved knowledge and perception. These individuals were also more likely to miss their ART doses making it less likely to have a suppressed VL. There is paucity of studies or data on socioeconomic status, the effect of this high mobility on VL suppression in KP. It is, therefore, important that healthcare providers emphasize the importance of ART medications, the side effects, the need for taking medications regularly, and their role in management of ART at every contact with the key population either physically or via phone communications. Whenever a VL result is received to be unsuppressed, EAC should commence at once to enable prompt correction of poor adherence, if issues with adherence is established to be the case. It is recommended that EAC should be done for 3 months before a repeat VL is done and if still unsuppressed, the patient is switched to another preferred regimen line. EAC is, therefore, particularly important for marginalized groups, such as sex workers, MSM, and male-to-female transgendered people. At the same time, it is important to empathize with KP and understand the issues they face while taking ART. KP may live in groups, and move regularly due to their profession, or may have their pills stolen by rogue elements at their place of work. Thus, their counseling session should not be the routine education/counseling session but rather an individual customized process, which evaluates their preparedness, knowledge, perception, and potential barriers in taking ART regularly [13]. Taking into consideration these challenges with the way KP live, it is recommended that promising means of delivery of EAC should be explored to provide the needed intervention to this group in an individual customized process. Different studies have reported different viral suppression rates among patients undergoing EAC sessions among KP and general populations. According to a study done in Zimbabwe [17], out of the 444 patients who had repeat VL after EAC after 3 months of previously unsuppressed VL, 201 (45.2%) had VL suppression with only 47.1% in those who underwent EAC and 33.9% in those who did not undergo EAC. However, Chi-square test still showed a level of significance at p-value = 0.05. Showing preference of EAC. Another study from Ethiopia [18] done within single cohort clearly reported that based on the paired sample test, there was a significant mean difference between VL at baseline and at the end of EAC sessions (mean difference=16,904, (95% CI: 9986-23,821; p-value<0.001) before and after EAC sessions. While a study in Zambia [19] demonstrated that out of 616 clients analyzed, there was an improvement in VL suppression following completion of EAC with a final outcome of 61% suppression. In Nigeria, a study on ‘’Effectiveness of Enhanced Adherence Counselling among unsuppressed key population individuals in South-South Nigeria’’ reported VL suppression of 97% among KP who were previously unsuppressed and had undergone EAC sessions [20]. These studies thus show the effectiveness of the WHO recommended EAC on achieving viral suppression among previously virally unsuppressed PLHIV/KPLHIV on ART.

Though EAC is effective on viral suppression, the EAC sessions are done monthly for 3 months through physical visit of patients to the facility. This constant visit is not tailored to suit the routine of an average person who have other non-medical and non-emergency matters to focus on. This is more so among KPs who are peculiar with mobility [3, 6].

 Considering that patients may find it burdensome to make monthly visits to the facility, which can sometimes affect the consistency and completion of the EAC sessions, creative approaches like the use of phone technology may be a way to deliver the EAC sessions without compromising the quality of information communicated, especially given that studies have shown the effectiveness of adherence counseling over the phone. For instance, findings from a study concluded that brief adherence support counseling delivered by phone demonstrates clinically meaningful improvements in ART adherence and HIV suppression [21]. According to other studies, behavioral counseling grounded in Self-Regulation Theory and delivered by phone has shown particular promise for improving ART adherence [19-21]. However, implementers of the phone approach should understand that although phone-delivered self-regulation counseling may improve adherence, studies repeatedly find patients faced with challenges to maintaining adherence over the long-term [21].

In this study, we, therefore, sought to introduce phone EAC sessions for KPs who are virally unsuppressed to adjust to challenges of non-compliance to commencement and completion of routine physical EAC session and assessed effectiveness of the phone EAC as compared to the routine physical EAC on achieving the expected viral suppression among this population.

## Materials and methods

Aim

This study aimed to compare the effectiveness of EAC administration via physical visit against EAC delivery via phone in terms of improving adherence and attaining viral suppression in KPs who were virally unsuppressed. Outcomes from the study will help determine if phone-based EAC is adopted or rejected as a substitute for monthly in-person EAC sessions, especially among KPs with high levels of mobility.

Study area and population

This study was done in Asaba, the capital city of Delta State, in the South-South geo-political zone of Nigeria. The 2006 National population census shows Delta State has a population of 4,112,445 (males: 2,069,309; females: 2,043,136). The HIV patients included in this study were drawn from three KP One-stop-shop (OSS) HIV treatment centers in Delta State.

Study design

This is a non-randomized prospective intervention study with intervention and control groups. The target population was virally unsuppressed KP who are on anti-retroviral treatment (ART) and was divided into two groups based on the modality of adherence counseling. Group A was the intervention group that received phone-based EAC, while Group B was the control group with traditional adherence counseling methods (in-person hospital visits). The study's design departed from randomization technique in favor of a selective stratification technique. This approach was deemed more suitable for achieving the study's objectives, with the resulting groupings reflecting individuals residing at varying distances from the facility, divided into those attending EAC via telephone and those attending in person.

Sample size

The study was conducted in three KP facilities (OSS) which have 92% of the total population of KP on ART in Delta state. Using the electronic medical record of the facilities, a total of 17,594 KPs living with HIV (KPLHIV) were active on treatment, out of which 7177 eligible for VL had results. This formed the total target population. Using raosfot sample size formula for the intervention study, with a target population of 7177, the sample size n and margin of error E are given by x= Z(c/100)2r(100-r), n= N x/((N-1)E2 + x) and E= Sqrt[(N - n)x/n(N-1)], where N is the population size, r is the fraction of responses of interest and Z(c/100) is the critical value, critical value for the confidence level c, a minimum sample size of 365 was selected. Making allowance of a 10% drop rate, 402 samples were selected as the minimum sample size for the study. However, for ethical reasons, all 484 patients who had VL result during the study period (April to August 2021) were included in the study as expected from WHO and national guidelines for EAC. Our sample size was, therefore, above the minimum sample size required to make inferences about the entire population of the study.

Sampling technique

Participants were selectively assigned into intervention and control groups using a simple stratification (ability vs. inability to physically attend EAC sessions in-person) sampling technique from the patients’ list on the electronic medical record; 242 in the intervention group (Group A) and 242 in the control group (Group B). Selected samples were either adults or emancipated minors/adolescents who consented to the study. All of the participants had access to their phone, and access to phones did not affect the grouping stratification. Groups were not randomized as those close to facilities were selected for physical EAC, and those in far-to-reach areas were selected for phone EAC to get a sample group that represents the objective of the study. 

Intervention approach

The patients were on standard-of-care ART from 2020 to 2021. Those with unsuppressed VL were selected, counseled, and consented to the study. The intervention group received EAC via phone conversation, while the control group received EAC via the routine physical visit to the facility. Both study groups received adherence assessment, adherence counseling, and adherence reinforcement monthly using a standardized CDC-EAC tool. This was provided by trained counselors, and the outcome was documented in EAC registries. By end of the third month after the EAC sessions, a repeat VL test was done, and pre-and-post-EAC VL results were analyzed for comparison and the significance of the intervention.

Data collection and data analysis

Patients with virally unsuppressed results by April 2021 received EAC sessions from trained counselors using structured EAC job aids either via phone conversation (for the intervention group) or physical conversation (for the control group). Commencement and completion of each monthly session were documented in the standardized national EAC register in prospective tracking until completion and report VL test and result documented and used to determine the next intervention approach by the clinical team.

Data and statistical analysis

Data recorded in the EAC registries were transferred into the facility's electronic medical record for individual patients and exported into Microsoft Excel for analysis. The export was analyzed using SPSS 24.0 statistical package (SPSS Inc., Chicago, USA). Means, percentages, and frequencies were used to present data as descriptive statistics for demographics, KP type, clinical, and ARV (anti-retro viral) treatment-related characteristics of participants. Whereas the Chi-square test and independent t-test were employed to assess differences in variables and statistically significant associations of the two types of EAC delivery with VL outcome at a p-value < 0.05.

Ethical considerations

Ethical approval for this study was obtained from the Delta State Ministry of Health, Asaba, Delta State. The participants were informed about the study, and informed consent was obtained. All the information collected from participants was treated with confidentiality.

*Inclusion criteria*: all patients were 15 years of age or older; all selected patients were HIV positive; patients who were included must have completed 6 months on ART; all selected patients were virally unsuppressed (VL > 1000 and above).

*Exclusion criteria*: pediatric populations were excluded; those with suppressed VLs were excluded from the study; patients who had not completed 6 months on ART were excluded; patients who started ART before 2020 were excluded.

## Results

**Figure 1:**
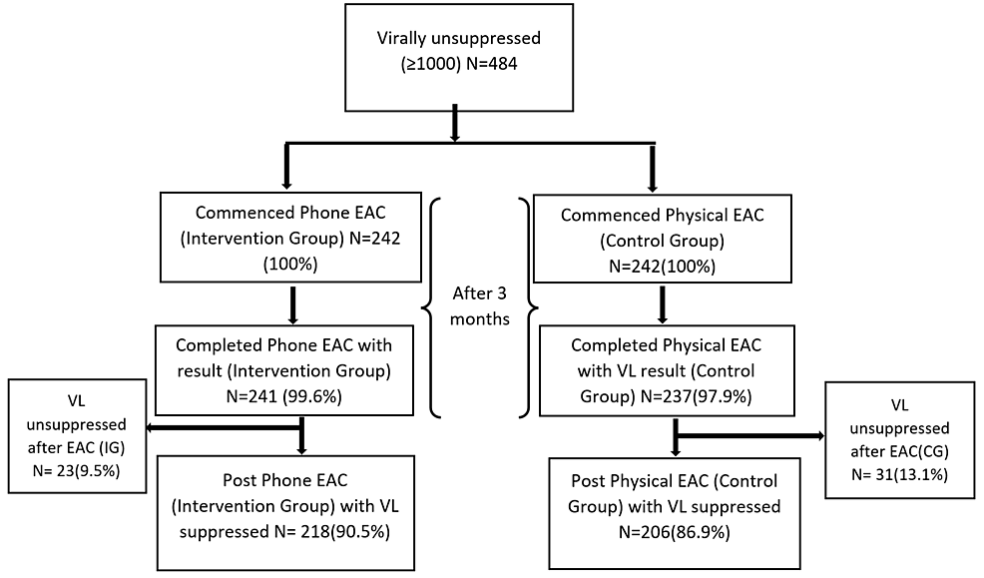
Inclusion and result cascade. VL, viral load; EAC, enhanced adherence counseling; IG, intervention group; CG, control group

Sociodemographic characteristic of virally unsuppressed KP patients of the 484 participants were included in this study: 242 in intervention group and 242 in control group. Figure 1 below describes the study cascade.

Demographic characteristics of participants

Table 1 below describes the demographic characteristics of participants. Majority of the participants were males, 423 (87%) with females 61(13%). Participants comprised mostly MSM (75%) explaining reasons why majority of participants are male, whereas 13% were FSW and 12% PWID. Participants had a mean age of 26 ± 2 with age range of 20-24 (36%), 25-29 (41%), and 30-34 (13%). Age 15-19 and >35 years made up less than 5% each. Some 13% were just 6 months on ART, majority (87%) were 6-12 months on ART, and only 2 persons were more than 12 months on ART. All participants were in WHO Stage 1 of HIV and all on WHO recommended optimized regimen in Nigeria of TLD/3TC/DTG.

**Table 1:** Demographic characteristics of participants. EAC, enhanced adherence counseling; KP, key population; FSW, female sex worker; MSM, men who have sex with men; PWID, people who inject drugs; ART, antiretroviral therapy; WHO STAGE, World Health Organization HIV staging; TDF-3TC-DTG, dolutegravir/lamivudine/tenofovir

Characteristics	Total n=484 Frequency (%)	Intervention group (Phone EAC) n=242 Frequency (%)	Control group (Physical EAC) n=242 Frequency (%)
SEX			
Female	61(12.6)	35(14.5)	26(10.7)
Male	423(87.4)	207(85.5)	216(89.3)
AGE			
15-19	21(4.3)	13(5.4)	8(3.3)
20-24	174(36.0)	86(35.5)	88(36.4)
25-29	198(40.9)	97(40.1)	101(41.7)
30-34	63(13.0)	30(12.4)	33(13.6)
≥35	28(5.8)	16(6.6)	12(5.0)
KP TYPE			
FSW	61(12.6)	35(14.5)	26(10.8)
MSM	363(75.0)	179(73.9)	184(76.0)
PWID	60(12.4)	28(11.6)	32(13.2)
DURATION ON ART			
6 months	61(12.6)	32(13.2)	29(12.0)
6-12 months	421(87.0)	209(86.4)	212(87.6)
>12 months	2(0.4)	1(0.4)	1(0.4)
WHO STAGE			
Stage 1	484(100.0)	242(100.0)	242(100.0)
REGIMEN TYPE			
TDF-3TC-DTG	484(100.0)	242(100.0)	242(100.0)

Commencement of EAC sessions among virally unsuppressed KP between the intervention and control groups

Table 2 above outlines participants’ characteristics and also shows pre-intervention characteristics between the intervention group (IG) and control groups (CG). No significant differences were observed in variables between the two study groups. There was consideration of relatively similar participants, thus there are no significant differences across the characteristics of participants between the two groups with p > 0.05.

**Table 2:** Commencement of EAC sessions among virally unsuppressed KP between the intervention and control groups. EAC, enhanced adherence counseling; KP, key population; FSW, female sex worker; MSM, men who have sex with men; PWID, people who inject drugs; ART, antiretroviral therapy; WHO STAGE, World Health Organization HIV staging; TDF-3TC-DTG. dolutegravir/lamivudine/tenofovir

Characteristics	Intervention group (Phone EAC) n=242 Frequency (%)	Control group (Physical EAC) n=242 Frequency (%)	Total n=484 Frequency (%)	X2	p value
SEX					
Female	35(14.5)	26(10.7)	61(12.6)	1.5194	0.22
Male	207(85.5)	216(89.3)	423(87.4)		
AGE					
15-19	13(5.4)	8(3.3)	21(4.3)	2.0086	0.73
20-24	86(35.5)	88(36.4)	174(36.0)		
25-29	97(40.1)	101(41.7)	198(40.9)		
30-34	30(12.4)	33(13.6)	63(13.0)		
35-39	16(6.6)	12(5.0)	28(5.8)		
KP TYPE					
FSW	35(14.5)	26(10.8)	61(12.6)	1.6634	0.44
MSM	179(73.9)	184(76.0)	363(75.0)		
PWID	28(11.6)	32(13.2)	60(12.4)		
DURATION ON ART					
6 months	32(13.2)	29(12.0)	61(12.6)	0.1689	0.92
6-12 months	209(86.4)	212(87.6)	421(87.0)		
>12 months	1(0.4)	1(0.4)	2(0.4)		
WHO STAGE					
Stage 1	242(100.0)	242(100.0)	484(100.0)	0	0
REGIMEN TYPE					
TDF-3TC-DTG	242(100.0)	242(100.0)	484(100.0)	0	0

Completion of EAC with result after 3 months

Table 3 below shows completion of EAC between the two groups. While there was higher completion rate at 99.6% (241 out of 242), completion in intervention group, and 97.9% (237 out of 242) completion for control group, there is no significant difference in completion rate between the two groups.

**Table 3:** Completion of EAC with result after 3 months. EAC, enhanced adherence counseling; KP, key population; FSW, female sex worker; MSM, men who have sex with men; PWID, people who inject drugs; ART, antiretroviral therapy; WHO STAGE, World Health Organization HIV staging; TDF-3TC-DTG, dolutegravir/lamivudine/tenofovir

Characteristics	Intervention group (Phone EAC) n=241(99.6%) Frequency(%)	Control group (Physical EAC) n=237(97.9%) Frequency(%)	Total n=478 (98.9%) Frequency(%)	X^2^	p value
SEX					
Female	35(14.5)	26(10.7)	61(12.8)	1.3544	0.24
Male	206(85.5)	211(89.3)	417(87.2)		
AGE					
15-19	12(5.4)	7(3.3)	19(4.0)	1.9902	0.66
20-24	86(35.5)	85(36.4)	171(35.8)		
25-29	97(40.1)	100(41.7)	197(41.2)		
30-34	30(12.4)	33(13.6)	63(13.2)		
≥35	16(6.6)	12(5.0)	28(5.9)		
KP TYPE					
FSW	35(14.5)	26(10.8)	61(12.8)	1.3885	0.49
MSM	178(73.9)	181(76.0)	359(75.1)		
PWID	28(11.6)	30(13.2)	58(12.1)		
DURATION ON ART					
6 months	32(13.2)	28(12.0)	60(12.6)	0.2122	0.91
6 -12 months	208(86.4)	208(87.6)	416(87.0)		
>12 months	1(0.4)	1(0.4)	2(0.4)		

Viral suppression before and after intervention among participants

Table 4 above shows significant difference before and after intervention in viral suppression across the different variables. With exception of those who were >12 months on ART having no difference in viral suppression after intervention, other variables from sex to age and type of KP all showed significant difference in achieving viral re-suppression after EAC ranging from 88% to 96.7% suppression.

**Table 4:** Pre and post-intervention table of EAC. Note: *statistically significant EAC, enhanced adherence counseling; KP, key population; FSW, female sex worker; MSM, men who have sex with men; PWID, people who inject drugs; ART, antiretroviral therapy; WHO STAGE, World Health Organization HIV staging; TDF-3TC-DTG, dolutegravir/lamivudine/tenofovir

	Before intervention N=484			After intervention N=478			
Variables	Total	Suppressed n=0(0%) Frequency (% suppression)	Unsuppressed n=484(100%) Frequency(% suppression)	Total	Suppressed n=424(88.7%) Frequency(% suppression)	Unsuppressed n=54(11.3%) Frequency(%)	Fisher's exact test p value
SEX							
Female	61	0(0.0)	61(100.0)	61	57(93.4)	4(6.6)	= 0.00001
Male	423	0(0.0)	423(100.0)	417	367(88.0)	50(12.0)	=0.00001
AGE							
15-19	21	0(0.0)	21(100.0)	19	18(94.7)	1(5.2)	= 0.00001
20-24	174	0(0.0)	174(100.0)	171	150(87.7)	21(12.3)	= 0.00001
25-29	198	0(0.0)	198(100.0)	197	173(87.8)	24(12.2)	= 0.00001
30-34	63	0(0.0)	63(100.0)	63	57(90.5)	6(9.5)	= 0.00001
>35	28	0(0.0)	28(100.0)	28	26(92.9)	2(7.1)	= 0.00001
KP TYPE							
FSW	61	0(0.0)	61(100.0)	61	57(93.4)	4(6.6)	= 0.00001
MSM	363	0(0.0)	363(100.0)	359	316(88.0)	43(12.0)	= 0.00001
PWID	60	0(0.0)	60(100.0)	58	51(87.9)	7(12.1)	= 0.00001
DURATION ON ART							
6 months	61	0(0.0)	61(100.0)	60	58(96.7)	2(3.3)	= 0.00001
6-12 months	421	0(0.0)	421(100.0)	416	366(88.0)	50(12.0)	= 0.00001
>12 months	2	0(0.0)	2(100.0)	2	0(0.0)	2(100.0)	1

Comparative analysis of viral suppression between the intervention and control groups at before and after intervention

Tables 5-6 below show significant difference in viral suppression between the two study groups before and after EAC sessions using physical delivery for control group and phone for intervention group. Before the intervention, both groups had unsuppressed VL at baseline therefore both groups had 0% viral suppression before intervention. After intervention, while the intervention group with phone EAC had 90.5% re-suppression, the control group with physical EAC had 86.9% re-suppression. 9.5% in intervention group, and 13.1% in control group remained unsuppressed.

**Table 5:** Statistical comparison group for viral suppression by frequency. Note: *statistically significant; VL, viral load

Variable	Intervention group (Phone EAC) Frequency (% suppression)	Control group (Physical EAC) Frequency (% suppression)	Total frequency (%)
Before intervention	242	242	484
VL suppressed n(%)	0(0.0)	0(0.0)	0(0.0)
VL unsuppressed n(%)	242(100.0)	242(100.0)	484(100.0)
After intervention	241	237	478
VL suppressed n(%)	218(90.5)	206(86.9)	424(88.7)
VL unsuppressed n(%)	23(9.5)	31(13.1))	54(11.3)

 

**Table 6:** Statistical comparison within groups for viral suppression rates by p-value. Note: *statistically significant EAC, enhanced adherence counseling

		Before intervention	After intervention		X^2^	p
Intervention group (Phone EAC)	Suppressed	0	218		396.0017	=0.00001*
	Unsuppressed	242	23			
Control group (physical EAC)	Suppressed	0	206		366.0821.	=0.00001*
	Unsuppressed	242	31			

Though there is significant difference before and after intervention of EAC within the two forms of EAC, (phone vs. in-person EAC), having higher suppression in the phone category as shown in Tables 5-6 above, Table 7 below shows that there is no statistical significant difference in viral suppression when phone and conventional physical EAC is compared.

**Table 7:** Statistical comparison between phone intervention EAC and in-person EAC. EAC, enhanced adherence counseling

	Intervention group (Phone EAC)	Control group (Physical EAC)	X^2^	p value
Suppressed before intervention	0 (0.00)	0 (0.00)	1.5082.	0.680371
Unsuppressed before Intervention	242 (242.00)	242 (242.00)		
Suppressed after intervention	218 (212.88)	206 (211.12)		
Unsuppressed after intervention	23 (27.11)	31 (26.89)		

## Discussion

Demographic characteristics of participants

With all 484 participants being virally unsuppressed, we assessed the demographic characteristics associated with unsuppressed VL among KP under study. Different studies [17-20] have different distributions by sex whereas our study showed majority were male than female. This could be because MSM comprises majority of the 17,802 key population clients in Delta State from the EMR as at the time of study. Thus from findings, the unsuppressed participants comprised mostly of MSM (75%) explaining reasons why majority of participants are male, whereas 13% were FSW and 12% PWID. Though studies have found the KP participants to vary from mean age of 19-31 years [17-20], our study participants are in mean age of 26 years, age that connotes independence and mobility as against the younger age that would have been under parents or guardian or older age that could have responsibility to spouse and family. Thus the innovative approach of delivering EAC through phone communication was explored.

Commencement and completion of EAC sessions among virally unsuppressed KP between the intervention and control groups

On commencement of EAC, all participants commenced on EAC using either of the two-delivery means. While almost all participants -- with exception of one person -- in the intervention group (Phone EAC) completed the sessions at 99.7%, five persons from the control group (who had physical EAC) could not complete the visits required to complete the EAC sessions leaving this group at 97.9% completion. This may align with concerns raised by Shrikala et al. [13], who opined that KP may live in groups and move regularly due to their profession or may have their pills stolen by rogue elements at their place of work. Thus, their counseling session should not be a routine education/counseling session but an individual customized process that evaluates their preparedness, knowledge, perception, and potential barriers to taking ART regularly. In furtherance, some other studies in Nigeria among KP [1, 6, 10] stated that though EAC is effective on viral suppression, the EAC sessions are done monthly for 3 months through the physical visit of patients to the facility thus, the constant visit is not tailored to suite the routine of an average person who has other non-medical and non-emergency matters to focus on which is more so among KPs who are peculiar with mobility [6, 11]. With our finding showing better completion of EAC among phone EAC group as against physical EAC, the former approach could be explored to encourage completion of these important counseling sessions that have been shown to improve viral suppression over time. This will also reduce the time spent visiting the clinic, including the cost incurred on transportation for a monthly visit which could be a barrier to completion. Moreover, considering the opportunity cost for time spent monthly in the clinic, the benefits of delivering the EAC through the phone, where available, should be explored.

Viral suppression before and after intervention among participants

Several studies globally have shown the effectiveness of the WHO-recommended EAC in achieving viral suppression among previously virally unsuppressed PLHIV/KPLHIV on ART [17-20]. Particularly a study among KP in Nigeria on ‘’Effectiveness of Enhanced Adherence Counselling among unsuppressed key population individuals in South-South Nigeria’’ reported VL suppression of 97% among KP who were previously unsuppressed and had undergone EAC sessions [20]. This finding is not far from our finding, which showed 90% suppression among KP who underwent phone EAC and 88% among those with physical EAC. Though the expected 95% suppression by UNAIDS was not achieved after EAC, further post-EAC intervention is expected to achieve the 95% mark. 

Comparing viral suppression between the intervention and control groups before and after the intervention

Though there are limited studies on phone-based EAC among PLHIV/KPLIV, there have been reports/studies on phone-based adherence counseling among patients on ART with reported improvement in adherence. Findings from Kalichman et al. [21] which concluded that brief adherence support counseling delivered by phone demonstrates clinically meaningful improvements in ART adherence and HIV suppression aligns with our finding, which found a significant difference in viral suppression after intervention especially using phone-based EAC which suppression skyrocketing from 0% to 90%. Yet another study agrees with our finding of the effectiveness of phone-based EAC by stating that behavioral counseling grounded in self-regulation theory delivered by phone has shown particular promise for improving ART adherence [21-22]. Phone-based EAC is thus proven as effective and, in our findings, slightly more effective than physical EAC in terms of completing EAC sessions and achieving viral suppression.

Study limitation

The study sample technique utilized a stratified selected method, which could have affected the study outcome. This sampling technique may have caused over-representation or under-selection of certain groups: the selection criteria for this study may have favored participants with a certain demographic or clinical profile, and thus the sample may not be generalized. Furthermore, this limitation may have caused sampling bias. For example, those who consented may be more motivated to adhere to phone counseling than those who do not volunteer.

## Conclusions

There is significant difference before and after intervention of phone and in-person EAC. Though there is higher suppression in the phone category, no statistical significant difference was seen in viral suppression when phone and conventional physical EAC is compared. Also, other findings from our study, when compared with other studies, demonstrated that EAC is effective in achieving viral suppression by up to 90% among previously virally unsuppressed KPs. Phone-based EAC has also proven effective and, in our finding, slightly more effective than the conventional physical EAC in terms of completion of EAC sessions and achieving viral suppression. More so as phone-based EAC addresses the challenges associated with a monthly physical visit for EAC, which include travel time spent to the facility, cost of transportation to the facility, and forgone alternative of other activities, the patients would have gainfully engaged in. Phone-based EAC, on the other hand, has the potential to address the challenges of high mobility seen with KPs that could affect the completion of EAC if restricted only to a monthly physical visit.

We, therefore, recommend acceptance of providing KPLHIV and even PLHIV with options of receiving EAC via phone on scheduled (flexible) dates. In addition, we recommend further study on qualitative analysis of challenges of phone-based EAC among KPLHIV and PLHIV and study on phone-based EAC potential to address challenges of high mobility seen with key population that could affect completion of EAC if restricted only to monthly physical visit.
